# The Interplay Between GLP-1-Based Therapies, the Gut Microbiome, and MASLD/MASH in Type 2 Diabetes Mellitus: A Narrative Review

**DOI:** 10.3390/biomedicines14040806

**Published:** 2026-04-01

**Authors:** Boris Dinkov, Diana Pendicheva-Duhlenska

**Affiliations:** 1 Department of Pharmacology and Toxicology, Medical University, 5800 Pleven, Bulgaria; pendicheva@gmail.com; 2 Working Group “Scientific Guidance and Expert Support for the Implementation of Pharmacogenomics in Clinical Practice”, Project BG-RRP-2.004-0003, Medical University, 5800 Pleven, Bulgaria; 3 Clinic of Endocrinology and Metabolic Diseases, University Hospital “Dr. Georgi Stranski”, 5800 Pleven, Bulgaria

**Keywords:** type 2 diabetes mellitus, MASLD, MASH, gut microbiome, GLP-1-based therapies, liraglutide, semaglutide, tirzepatide, dulaglutide, gut–liver axis

## Abstract

GLP-1-based drugs are approved for the treatment of type 2 diabetes mellitus (T2DM) and obesity. Metabolic dysfunction-associated steatotic liver disease (MASLD) affects more than 60% of patients with T2DM, and the gut microbiome plays a critical role in its pathogenesis. The gut–liver axis represents a key mechanistic link between dysbiosis and hepatic steatosis. A narrative literature review was conducted using PubMed, Scopus, and ClinicalTrials.gov (2015–2026). Search terms included “GLP-1 receptor agonist,” “microbiome,” “MASLD,” “MASH,” “NAFLD,” “NASH,” “liraglutide,” “semaglutide,” “tirzepatide,” “dulaglutide,” and “exenatide.” Of 363 identified articles, 330 were excluded due to duplication or non-relevant study design; 33 studies (18 preclinical, 15 clinical) were included. In preclinical models, liraglutide demonstrated normalization of the *Firmicutes*/*Bacteroidetes* ratio and increased *Bifidobacterium* and *Lactobacillus* spp., while tirzepatide significantly reduced hepatic steatosis and increased *Akkermansia* abundance in diabetic mice. Semaglutide improved gut barrier integrity, increased *Alloprevotella* and *Alistipes*, and ameliorated MASLD in murine models. In clinical studies, tirzepatide achieved MASH resolution in 44–62% of patients in the phase 2 SYNERGY-NASH trial. In August 2025, the FDA approved semaglutide for MASH with fibrosis based on the Phase 3 ESSENCE trial. A recent longitudinal study in T2DM patients showed that baseline microbiome composition predicted glycemic response to semaglutide, without significant changes in microbiome diversity. In conclusion, GLP-1-based therapies demonstrate consistent preclinical associations with gut microbiome modulation and reduction in hepatic steatosis. Baseline microbiome composition has been suggested as a potential predictor of treatment response, supporting a personalized approach to MASLD management and warranting future clinical studies.

## 1. Introduction

The past two decades have been marked by alarming data regarding the global burden of diabetes mellitus. According to the 11th edition of the IDF Diabetes Atlas (2026), in 2024, diabetes affected 11.11% of the global adult population aged 20–79 years, corresponding to 589 million people. Projections indicate that prevalence will reach 12.96% (853 million people) by 2050, representing a 44.82% increase [1]. Type 2 diabetes mellitus (T2DM) is a chronic metabolic condition characterized by hyperglycemia resulting from impaired insulin secretion, impaired insulin action, or both [2]. According to current concepts, T2DM is defined by a defect in insulin secretion occurring in the setting of insulin resistance. The disease is associated with both acute and chronic complications, which constitute major causes of morbidity and mortality [3].

Nonalcoholic fatty liver disease (NAFLD), now known as metabolic dysfunction-associated steatotic liver disease (MASLD), is the most prevalent chronic liver disease worldwide, affecting approximately 30% of the adult population [4] and more than 60% of individuals living with T2DM [5].

The reclassification from NAFLD/NASH to MASLD/MASH reflects the growing recognition that metabolic dysfunction is central to the pathogenesis and outcomes of this disease. Historically, the term NASH was first described in 1980, followed by the introduction of MAFLD in 2020 as a positive, metabolic criteria-based definition. In December 2023, a multinational hepatology consensus proposed the current terminology, MASLD and MASH, better capturing the metabolic underpinnings of the disease while avoiding stigmatizing terminology [6,7]. Throughout this review, the former NAFLD/NASH terminology is retained when referring to the original study populations and endpoints.

The present review aims to explore the gut microbiome as a possible mediator of the effects of GLP-1-based therapies on MASLD and metabolic dysfunction-associated steatohepatitis (MASH) in patients living with T2DM. In this context, current advances in the treatment of MASLD and MASH are presented. Relevant preclinical and clinical evidence is discussed, along with the role of lifestyle interventions in combination with therapeutic strategies. In this way, the present review provides an integrated and contemporary perspective on the interaction between GLP-1-based therapies, the gut microbiome, and MASLD in the setting of T2DM, within a rapidly evolving therapeutic landscape.

## 2. Methods

A narrative literature review was conducted using the PubMed, Scopus, and ClinicalTrials.gov databases, covering the period from 2015 to 2026. The search keywords included “GLP-1 receptor agonist,” “microbiome,” “fatty liver disease,” “nonalcoholic steatohepatitis,” “MASLD,” “MASH,” “NAFLD,” “NASH,” “exenatide,” “tirzepatide,” “liraglutide,” “dulaglutide,” and “semaglutide”. This time frame was selected to reflect the contemporary therapeutic landscape, as the majority of GLP-1 receptor agonists relevant to the topic were approved or gained clinical relevance after 2015, and the most mechanistically relevant data linking these therapies to gut microbiome modulation and hepatic outcomes have been published predominantly within this period.

Studies were included if they reported original data on GLP-1-based therapies and at least one of the following outcomes: gut microbiome composition, hepatic steatosis, inflammation, or fibrosis. Studies without primary data were excluded, as were studies lacking sufficient methodological detail, defined as the absence of a clearly described intervention protocol, microbiome profiling methodology, or reportable hepatic outcome measures. A total of 363 articles were identified. Of these, 330 were excluded due to duplication or non-relevant study design (case reports, editorials, letters, or insufficient methodological detail). Thirty-three studies reporting original preclinical and clinical data on GLP-1-based therapies, gut microbiome composition, and hepatic outcomes were included in the narrative synthesis—18 preclinical studies and 15 clinical studies—alongside additional references providing contextual and mechanistic background. Given the limited number of studies directly addressing this topic and the substantial heterogeneity of available data derived from different animal models, pharmacological agents, and microbiome analysis methods, a narrative review was selected as the most appropriate methodological approach.

## 3. Pathophysiology of MASLD and MASH

MASLD is considered the hepatic manifestation of metabolic syndrome, with a progressive course that may advance to steatohepatitis, liver fibrosis or cirrhosis, and an increased risk of hepatocellular carcinoma [8]. The diagnosis of MASLD is established in the presence of hepatic steatosis and at least one cardiometabolic risk factor (overweight/obesity, dysglycemia, hypertension, hypertriglyceridemia, and/or low HDL cholesterol), in the absence of significant alcohol consumption [7]. Accumulation of lipids in hepatocytes may lead to lipotoxicity, which triggers mitochondrial dysfunction, oxidative stress, and cell death [9]. Hepatocyte death activates hepatic stellate cells, which differentiate into myofibroblasts and produce excessive amounts of extracellular matrix [8]. MASH represents the progressive inflammatory stage of MASLD, and it is histologically defined by the presence of ≥5% hepatic steatosis with lobular inflammation and hepatocyte ballooning (i.e., steatohepatitis), with or without fibrosis, in individuals meeting MASLD criteria [6].

The progressive nature of MASLD, with its potential to advance to MASH, cirrhosis and hepatocellular carcinoma, underscores the urgent need for effective pharmacological interventions beyond lifestyle modification.

### Therapeutic Interventions in MASLD and MASH

Currently, the cornerstone of MASLD and MASH management focuses on sustained lifestyle modifications through dietary interventions aimed at achieving a durable caloric deficit, combined with regular physical activity. In patients with MASLD, a 5% reduction in body weight may lead to reversal of steatosis, while weight loss exceeding 10% is associated with fibrosis regression.

On 15 August 2025, the FDA approved semaglutide for the treatment of MASH, making it, to date, the first and only GLP-1RA with this indication [10,11]. ESSENCE is a phase 3 double-blind, randomized, placebo-controlled clinical trial evaluating semaglutide 2.4 mg administered subcutaneously once weekly in adults with MASH and fibrosis stages F2 and F3. In this trial, 62.9% of participants in the semaglutide group achieved a reduction in liver-related inflammation without worsening, and even with regression, of fibrosis, compared with a 34.3% improvement in the placebo group [11]. However, further studies are needed to elucidate the potential relationship between the pathogenesis and progression of MASLD, MASH, and the gut microbiome.

The FDA approval of semaglutide for MASH with fibrosis marks a paradigm shift toward metabolism-targeted pharmacotherapy, positioning GLP-1-based therapies at the forefront of MASLD management.

## 4. GLP-1-Based Therapy

GLP-1 and GIP are incretin hormones secreted by the gastrointestinal tract (GIT) during food intake and contribute to glucose-dependent insulin secretion [12]. Native GLP-1 is synthesized by L-cells in the distal ileum, while GIP is secreted by K-cells in the duodenum and proximal jejunum. These two intestinal hormones are responsible for the so-called incretin effect, accounting for approximately 50–70% of insulin secretion in healthy individuals following oral glucose intake [13].

Over the past several years, a revolution has been observed in the treatment of type 2 diabetes mellitus and obesity. Novel therapeutic agents with different mechanisms of action have been developed, with a particular focus on GLP-1-based therapies. Contemporary agents within this class include GLP-1 receptor agonists and co-agonists targeting GLP-1, GIP, and the glucagon receptor. Their use is associated with favorable effects on body weight and insulin sensitivity, improvement in lipid profiles, reduction in atherogenic dyslipidemia, and lowering of arterial blood pressure [14].

### 4.1. GLP-1 Receptor Agonists

GLP-1 receptor agonists induce satiety and reduce appetite, leading to decreased caloric intake and significant weight loss [15,16]. GLP-1RAs improve β-cell function and, in preclinical and in vitro models, promote β-cell proliferation, inhibit apoptosis, and attenuate dedifferentiation; however, direct evidence for these effects in humans in vivo remains limited [17,18].

Exenatide is the first approved GLP-1RA, available in two formulations: immediate-release (administered subcutaneously twice daily) and extended-release (once weekly). In the EXSCEL trial, once-weekly exenatide demonstrated cardiovascular safety but did not achieve superiority over placebo [19]. Exenatide extended-release reduces HbA1c and body weight by 2.3–3.7 kg, primarily through reduced food intake via enhanced satiety and appetite suppression [20].

Liraglutide is a GLP-1RA approved for the treatment of T2DM and obesity, administered subcutaneously once daily. In the LEADER trial, liraglutide at a dose of 1.8 mg daily demonstrated a significant reduction in cardiovascular events in T2DM patients at high cardiovascular risk [21]. At a dose of 3.0 mg, liraglutide achieves clinically meaningful reductions in body weight, as an adjunct to diet and exercise, as demonstrated in the SCALE trials [22,23].

Semaglutide is a next-generation GLP-1RA with higher receptor affinity, administered subcutaneously once weekly or orally once daily. In the STEP trial, semaglutide at a dose of 2.4 mg achieved a mean body weight reduction of up to 15–17%, surpassing the effect of liraglutide [24]. In addition, semaglutide demonstrated cardiovascular and renal benefits in the SUSTAIN-6, SOUL, and FLOW trials [25,26,27]. On 15 August 2025, semaglutide was approved by the FDA as the first GLP-1RA for the treatment of MASH with fibrosis [10] (see Section 3).

Dulaglutide is a long-acting GLP-1RA administered subcutaneously once weekly. The REWIND trial demonstrated a significant reduction in cardiovascular events in patients with T2DM with or without prior cardiovascular disease [28]. At higher doses (3.0 mg and 4.5 mg weekly), dulaglutide achieved greater reductions in HbA1c and body weight compared with the standard 1.5 mg dose [29].

Collectively, GLP-1 receptor agonists represent a well-established drug class with proven cardiovascular, renal, and hepatic benefits in T2DM.

### 4.2. Dual GLP-1 and GIP Receptor Agonists

Tirzepatide is the first dual agonist of the GLP-1 and GIP receptors, administered subcutaneously once weekly. Concurrent activation of both receptors provides a synergistic effect on insulin secretion, appetite regulation, and body weight reduction, while GIP receptor stimulation additionally improves lipid metabolism in adipose tissue. In the SURPASS trial, tirzepatide demonstrated a reduction in HbA1c of 2–2.5%, exceeding the effect observed with semaglutide [30]. The SURMOUNT trial showed weight loss of up to 22.5% at a dose of 15 mg—the most substantial reduction among currently approved anti-obesity medications [31].

Phase 2 of the SYNERGY-NASH trial demonstrated that tirzepatide (5 mg, 10 mg, or 15 mg) significantly reduced MASH without worsening of fibrosis in 44–62% of patients, compared with 10% in the placebo group. The results showed superior improvements in liver histology and fibrosis at week 52 [11].

The dual mechanism of action of tirzepatide positions it as a particularly promising agent for the treatment of MASLD and MASH in T2DM.

### 4.3. Tolerability and Safety of GLP-1RAs and GLP-1RA/GIP Agonists

The most common adverse effects of GLP-1 receptor agonists are gastrointestinal symptoms, which are generally mild to moderate in severity and tend to diminish over time. GLP-1 receptor agonists and dual GLP-1/GIP agonists (tirzepatide) demonstrate comparable tolerability profiles, with gastrointestinal symptoms representing the most frequent adverse reactions in both drug classes [32,33,34]. In a direct comparative study, tirzepatide showed lower absolute rates of nausea (28% vs. 44%) and vomiting (13% vs. 24%) compared with semaglutide 2.4 mg [35]. Accordingly, treatment discontinuation due to gastrointestinal events was lower with tirzepatide (2.7%) than with semaglutide (5.6%) [35]. Serious gastrointestinal events, including pancreatitis, biliary disease, and intestinal obstruction, were comparable across dulaglutide, semaglutide, and tirzepatide in a large cohort study involving more than 130,000 patients [36]. From a mechanistic perspective, GIP receptor agonism may modulate the gastrointestinal effects of GLP-1, thereby contributing to the improved tolerability of tirzepatide [37]. In both drug classes, slow dose titration remains the key strategy for minimizing adverse effects.

The favorable tolerability profile of GLP-1-based therapies supports their long-term use in patients with T2DM and MASLD.

### 4.4. Multi-Agonists of GLP-1/GIP/Glucagon Receptors Under Development

In parallel with approved therapies, molecules simultaneously targeting three receptors within the incretin axis are actively being developed. It has been established that the anorexigenic effect and increased energy expenditure are mediated through activation of the GLP-1 receptor and the glucagon receptor (GCGR), respectively [38,39]. Consequently, dual and triple agonists of the GLP-1 receptor, GIP receptor, and GCGR represent a promising new therapeutic option for patients with type 2 diabetes mellitus and/or obesity, offering greater weight reduction and glucose-lowering effects compared with single GLP-1 receptor agonists [38].

The mechanism by which activation of the glucagon receptor reduces food intake is not fully elucidated. It is hypothesized that reduced food intake observed in both rodents and humans results from activation of glucagon receptor–expressing vagal afferent pathways from the liver to the hypothalamus, as well as from direct effects on glucagon receptors in the arcuate nucleus of the hypothalamus [40]. Growing evidence suggests that GLP-1-based therapies may improve liver function, reduce chronic inflammation, sleep apnea, and potentially degenerative bone disorders and cognitive decline [41].

Novel peptide-based incretin therapies currently under development include a long-acting glucagon receptor agonist LY3324954 [42], dual GLP-1 and GCGR agonist survodutide [43], and the triple GLP-1/GIP/GCGR agonist retatrutide [44]. In addition to GLP-1 agonists, amylin analogs such as cagrilintide and the single-molecule dual GLP-1 and amylin receptor agonist amicretin are also being investigated in synergy with GLP-1RAs for their potential effects on body weight and metabolic parameters [41].

The growing interest in the potential of GLP-1-based therapies to influence not only glycemic control and body weight, but also MASLD, MASH, and the associated liver fibrosis, raises the question of whether these effects may be at least partially mediated through modulation of the gut microbiome.

## 5. Significance of the Gut Microbiome for Metabolic Health

The human gut microbiome represents a complex community of commensal bacteria (approximately 95%) and other microorganisms, including viruses and fungi, inhabiting the human gastrointestinal tract (GIT), with the highest density located in the colon [45]. Over the past decade, results from more than tens of thousands of studies have been published, providing data on the composition and physiological functions of the gut microbiome, and its association with the pathogenesis of numerous diseases, including type 2 diabetes mellitus, cardiovascular diseases, depression, Parkinson’s disease, colorectal cancer, and chronic inflammatory bowel diseases.

Contemporary research encourages the investigation of the relationships between diet, health, and microbial diversity, as well as the development of personalized nutritional strategies and health recommendations based on the gut microbiome. The human GIT contains from several tens to approximately one hundred trillion bacteria, predominantly belonging to several major phylotypic groups: the phylum *Bacteroidetes* (genera *Bacteroides* and *Prevotella*), the phylum *Firmicutes* (genera *Clostridium*, *Enterococcus*, and *Lactobacillus*), and, to a lesser extent, the phyla *Actinobacteria* (genus *Bifidobacterium*) and *Proteobacteria* (genera *Helicobacter* and *Escherichia*).

Numerous studies focus on their local and systemic effects, which include suppression of pathogenic bacteria and participation in the maintenance of host homeostasis through regulation of multiple endocrine and metabolic processes. The gut microbiota is capable of increasing nutrient and energy absorption depending on the composition of the consumed diet, thereby affecting energy homeostasis. Multiple mechanisms are thought to be involved in this complex process, including increased absorption of monosaccharides; fermentation of indigestible dietary polysaccharides (fibers) into short-chain fatty acids (SCFAs); stimulation of hepatic lipogenesis with increased expression of key lipogenic enzymes; utilization of SCFAs absorbed by the intestinal mucosa as an energy source; increased lipid storage in adipocytes through enhanced lipoprotein lipase activity; and reduced fatty acid oxidation in the liver and skeletal muscle [45].

Short-chain fatty acids, primarily acetate, propionate, and butyrate, exert multiple physiological effects on host metabolism. Butyrate serves as the primary energy source for colonocytes and maintains intestinal barrier integrity through upregulation of tight junction proteins [46]. Propionate is transported via the portal vein to the liver, where in vitro studies show it suppresses gluconeogenesis through activation of AMP-activated protein kinase (AMPK) and GPR43 signaling [47]. However, in vivo human data suggest propionate may paradoxically increase glucagon and fatty acid-binding protein 4 (FABP4) production, impairing insulin action through activation of the sympathetic nervous system [48]. SCFAs reduce hepatic triglyceride accumulation by inhibiting SREBP-1c-mediated lipogenesis and enhance fatty acid oxidation through PPARγ-dependent switching from lipogenesis to fat oxidation (rather than direct PPARα activation), decreasing PPARγ expression and activity, which increases mitochondrial UCP2 and stimulates oxidative metabolism via AMPK [49]. These seemingly divergent findings suggest that the metabolic effects of SCFAs, particularly propionate, may be context-dependent and influenced by factors such as dose, host metabolic status, and microbiome composition.

### 5.1. Significance of the Gut Microbiome in MASLD

The liver and the intestine maintain bidirectional communication through their anatomical connection, whereby the portal vein transports nutrients, toxins, and microbial products to the liver, while the liver secretes bile that regulates the gut microbiota, as well as hormones such as cholecystokinin, fibroblast growth factor 19 (FGF-19), and insulin-like growth factor [50,51,52]. Bile acids synthesized by hepatocytes from cholesterol facilitate digestion in the small intestine and act as signaling molecules through the farnesoid X receptor (FXR) and the G protein-coupled bile acid receptor (TGR5, GP-BAR1) [53], thereby regulating glucose and lipid metabolism, as well as their own synthesis via fibroblast growth factor 19 (FGF-19). At the same time, bile acids interact bidirectionally with the gut microbiota, modulating its composition while themselves being metabolized by it.

It is well established that the gut microbiome plays a critical role in the pathogenesis and progression of MASLD. This role is exerted through regulation of intestinal permeability, alteration of luminal bile acid metabolism and dietary substrates, primarily non-digestible carbohydrates (resistant starch, non-starch polysaccharides, oligosaccharides), proteins, and phytochemicals, which are fermented by the gut microbiota, yielding short-chain fatty acids (acetate, propionate, butyrate) and other metabolites that influence host metabolism and hepatic function [54], as well as through increasing the production of lipoprotein lipase, endogenous alcohol (ethanol), and toxic compounds [55]. In individuals with MASLD, dysbiosis reduces the production of antimicrobial peptides and tight junction proteins between intestinal epithelial cells, while simultaneously altering the number of immune cells in the lamina propria. This increases intestinal permeability and disrupts regulation of the gut–liver axis [8]. Disruption of the epithelial and vascular barriers leads to translocation of microbial components, including bacterial toxins, into the portal and systemic circulation. Lipopolysaccharide (LPS), an endotoxin, is recognized by Toll-like receptor 4 (TLR4) on hepatic Kupffer cells, activating myeloid differentiation primary response 88 (MyD88) and interferon regulatory factor 3 (IRF3), resulting in a proinflammatory response with secretion of tumor necrosis factor alpha (TNF-α) [56].

Progression from steatosis to steatohepatitis and fibrosis involves activation of the NLRP3 inflammasome (NOD-, LRR-, and pyrin domain-containing protein 3) and production of damage-associated molecular patterns (DAMPs), which activate caspase-1 and interleukin-1β (IL-1β) and stimulate hepatic stellate cells toward fibrogenesis. Beyond direct injury, translocated bacteria also exert hepatic effects through the production of microbial metabolites.

Moreover, specific microbiome-derived metabolites promote disease progression through distinct mechanisms. Ethanol produced by gut bacteria induces hepatic oxidative stress and inflammation, while trimethylamine N-oxide (TMAO) promotes lipid accumulation and cardiovascular risk. Lactate, derived from microbial fermentation, contributes to metabolic dysregulation and hepatic lipid accumulation. These metabolites contribute to hepatocellular injury and gut barrier disruption, which secondarily reduces bile acid synthesis and enterohepatic circulation, thereby diminishing FXR activation—a key regulatory pathway for glucose and lipid homeostasis.

**Figure 1:**
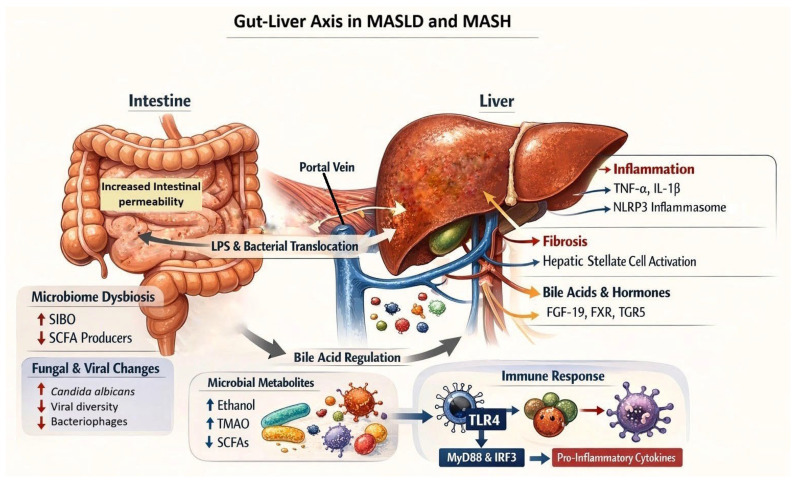
The gut–liver axis in MASLD and MASH: pathophysiological mechanisms linking gut microbiome dysbiosis to hepatic steatosis, inflammation, and fibrosis. Abbreviations: SIBO—small intestinal bacterial overgrowth; SCFA—short-chain fatty acids; TMAO—trimethylamine N-oxide; LPS—lipopolysaccharide; TLR4—Toll-like receptor 4; MyD88—myeloid differentiation primary response 88; IRF3—interferon regulatory factor 3; TNF-α—tumor necrosis factor alpha; IL-1β—interleukin-1β; NLRP3—NOD-, LRR-, and pyrin domain-containing protein 3; FGF-19—fibroblast growth factor 19; FXR—farnesoid X receptor; TGR5—G protein-coupled bile acid receptor.

Additionally, LPS induces hepatic inflammation through TLR4 activation. Short-chain fatty acids (SCFAs), on the other hand, possess anti-inflammatory properties that may slow the progression of MASLD [57]. Alterations in the composition and activity of the gut microbiome may lead to impaired production of these microbial metabolites, thereby contributing to inflammation, insulin resistance, hepatic lipid accumulation, and liver fibrosis. The key pathophysiological mechanisms underlying the gut–liver axis in MASLD and MASH are illustrated in Figure 1.

The bacterial microbiome in MASLD is characterized by several key alterations. Small intestinal bacterial overgrowth (SIBO) is observed in up to 35% of patients with MASLD, with prevalence increasing to 47.1% in MASH, and predominance of *Escherichia coli* and *Staphylococcus aureus* in small intestinal aspirates [58,59]. A meta-analysis of 54 studies involving 8894 participants demonstrated reduced abundance of anti-inflammatory genera such as *Alistipes*, *Blautia*, and *Faecalibacterium*, along with increased abundance of potentially pathogenic taxa including *Fusobacteriaceae*, *Enterococcaceae*, and *Escherichia* [60]. These findings were confirmed by a systematic review of 28 studies including 3566 participants, which demonstrated decreased relative abundance of SCFA-producing bacteria (*Ruminococcus*, *Faecalibacterium*, *Coprococcus*) and increased abundance of *Escherichia* [61,62]. As MASLD progresses from steatosis to cirrhosis (F4), a stepwise increase in *E. coli* and a reduction in *Eubacterium rectale*, *Faecalibacterium prausnitzii*, and *Dorea longicatena* have been observed using metagenomic sequencing [63,64].

Beyond the bacterial microbiome, the fungal microbiota (mycobiome) is also altered in MASLD. Reduced fungal diversity has been documented in patients with MASLD compared with controls, with a relative decrease in *Saccharomyces cerevisiae* and an increase in *Mucor ambiguous* [65]. In patients with MASH or advanced fibrosis, a characteristic fecal mycobiome has been identified with increased abundance of *Candida albicans* and other taxa, and oral antifungal treatment with amphotericin B has reduced steatohepatitis and fibrosis in a humanized mouse model (germ-free mice colonized with fecal microbiota from patients with MASH and fed a Western diet for 20 weeks) [66]. Higher systemic IgG antibodies against *C. albicans* in patients with advanced liver fibrosis suggest translocation of fungal components to the liver through a disrupted intestinal barrier [66].

Regarding the virome, histological severity of MASLD has been associated with reduced viral diversity and a lower proportion of bacteriophages in the gut virome, including a decrease in specific taxa such as *Lactococcus* phages in advanced fibrosis [67]. Consumption of even low to moderate amounts of alcohol in patients with MASLD significantly affects the gut virome, with viral diversity in this group comparable to that observed in alcohol-associated liver disease (ALD) [68]. Further mechanistic studies are required to clarify the causal role of the virome in MASLD progression.

Several animal models of diabetes are widely used to investigate the mechanisms underlying metabolic dysfunction, diabetic complications, and the development of novel pharmacological and therapeutic interventions. Similarities in gut microbiome composition have been observed between rats and humans, with predominance of the phyla *Bacteroidetes* and *Firmicutes* in both species, although interindividual variability of the microbiota is substantially higher in humans than in rats. Specificity can be observed at the genus and species level. Available data indicate that rats are representative of the human gut microbiota [69], making them a suitable model for studying the human microbiome and metabolism. This underpins investigations into the functional roles of the microbiome, including carbohydrate degradation, SCFA synthesis, and immune interactions. Of particular importance is the identification of bacteria of the genus *Akkermansia*, such as *A. muciniphila*, which has been identified as one of several microbial taxa associated with metabolic health [70].

Experimental studies of the gut microbiome in rats with induced T2DM demonstrate increased levels of *Firmicutes* and *Proteobacteria*, whereas healthy rodents show higher levels of *Bacteroidetes*. The predominant bacterial groups (over 90%) in such experimental models include *Firmicutes*, *Bacteroidetes*, *Proteobacteria*, *Actinobacteria*, *Fusobacteria*, *Tenericutes*, and *Verrucomicrobia*. At present, the scientific literature contains limited data regarding the relationship between GLP-1 receptor agonist therapy, modulation of the gut microbiome, and liver function.

Taken together, these findings establish the gut–liver axis as a link between dysbiosis and hepatic disease progression.

### 5.2. GLP-1 and the Gut Microbiome

The gut microbiota does not directly metabolize or utilize GLP-1 receptor agonists. A bidirectional relationship exists between GLP-1 receptor agonists and the gut microbiome, whereby the drugs modulate microbiome composition while microbial metabolites have been shown to stimulate endogenous GLP-1 secretion [71,72].

The mechanisms of interaction include the following: GLP-1 receptor agonists alter gut microbiota composition through several pathways, such as activation of the sympathetic nervous system, leading to changes in the intestinal environment; modulation of intestinal intraepithelial lymphocytes expressing GLP-1 receptors, which are essential for the full effect of GLP-1 agonists on the microbiota; and anti-inflammatory action, which indirectly influences microbial composition [73,74,75]. The reverse direction of interaction is equally important. The gut microbiota modulates endogenous GLP-1 secretion through microbial metabolites such as short-chain fatty acids and bile acid derivatives, which stimulate L-cells to produce GLP-1. Importantly, dietary changes and weight loss induced by GLP-1 receptor agonists are likely the primary drivers of the observed microbiome alterations, rather than direct metabolism of the drugs by bacteria [71,75,76].

**Figure 2:**
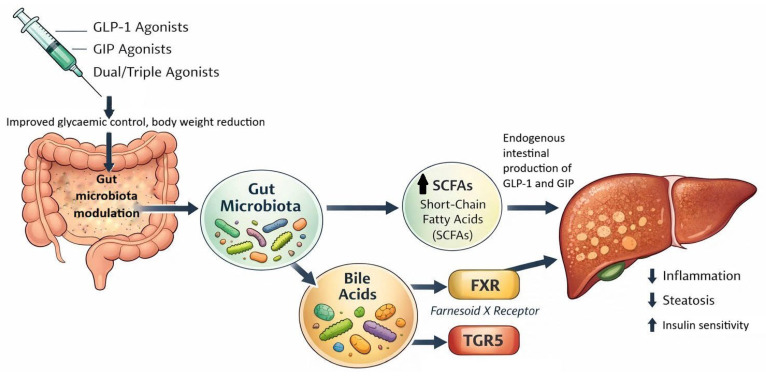
Putative gut microbiome-mediated mechanisms by which GLP-1-based therapies affect hepatic metabolism. Abbreviations: GLP-1RA—glucagon-like peptide-1 receptor agonist; GLP-1/GIP RA—dual glucagon-like peptide-1 and glucose-dependent insulinotropic polypeptide receptor agonist; GLP-1—glucagon-like peptide-1; GIP—glucose-dependent insulinotropic polypeptide; SCFA—short-chain fatty acids; FXR—Farnesoid X receptor; TGR5—G-protein-coupled bile acid receptor.

Increasing evidence suggests that GLP-1RAs modulate the composition of the gut microbiome, which may contribute to their metabolic effects in MASLD (Figure 2). A systematic review of 38 studies demonstrated that liraglutide promotes the growth of genera associated with beneficial metabolic functions, including increased abundance of *Akkermansia muciniphila*, *Lactobacillus*, and *Faecalibacterium prausnitzii,* species with proven anti-inflammatory effects and positive influence on the intestinal barrier. Of particular importance is *Lactobacillus reuteri*, which, besides being increased by GLP-1RA therapy, itself stimulates endogenous intestinal GLP-1 secretion, creating a potential positive feedback loop [77]. However, longitudinal cohort studies with standardized microbiome profiling and integration of multi-omics approaches are required to determine whether these microbial changes represent a primary therapeutic mechanism or a secondary effect of metabolic improvement in MASLD/MASH.

The bidirectional relationship between GLP-1 receptor agonists and the gut microbiome suggests that microbiome modulation represents an indirect component of their therapeutic mechanism.

### 5.3. The Gut Microbiome as a Possible Mediator of GLP-1-Based Therapies and MASLD

Several studies on liraglutide show normalization of the Firmicutes/Bacteroidetes ratio and increased abundance of probiotic *Bifidobacterium* and *Lactobacillus* spp. [55,78]. Results from a clinical study by Ying et al. (2023) [79] demonstrated that liraglutide reduces body weight and plasma glucose levels, improves lipid metabolism, decreases inflammation, and enhances liver function. However, its effect on gut microbiome composition was relatively modest.

In contrast, a study by Chen et al. (2025) [80], including 15 Chinese patients with T2DM treated with semaglutide for 12 weeks, demonstrated measurable changes in microbial composition—reduction in *Firmicutes* and increase in *Bifidobacterium*, accompanied by metabolomic changes in 362 differentially expressed metabolites. These findings suggest that semaglutide is associated with modest modulation of gut microbiome composition, likely mediated through improved glycemic control and metabolic changes rather than direct pharmacological action on the microbial community, although the small sample size limits the generalizability of the findings.

In another study using an experimental diabetes model, the dual GLP-1/GIP receptor agonist tirzepatide significantly reduced high-fat diet/streptozotocin (HFD/STZ)-induced hepatic steatosis by modulating the gut microbiota and bile acid metabolism. Tirzepatide intervention increased the abundance of *Akkermansia* in the gut microbiome [81].

In a recent study involving 29 mice with HFD-induced MASLD treated with tirzepatide for 20 weeks, the intervention significantly reduced serum levels of ALT and AST, as well as hepatic triglycerides (TG) and total cholesterol (TC), demonstrating its efficacy in MASLD treatment [82]. Metabolomic and proteomic analyses showed that tirzepatide decreases fatty acid uptake by downregulating CD36 and Fabp2/4, improves mitochondrial–lysosomal function via up-regulation of Lamp1/2, and enhances cholesterol efflux by increasing expression of Hnf4a, Abcg5, and Abcg8 [82]. Key preclinical studies examining the effects of GLP-1-based therapies are summarized in Table 1.

Recent data from an experimental diabetes model in mice demonstrate that semaglutide improves beta-cell function through modulation of the gut microbiome—it significantly decreases members of the genera *Firmicutes*, *Actinobacteriota*, and *Lactobacillus*, while simultaneously increasing the abundance of *Bacteroides* and *Muribaculaceae* and the production of SCFAs [93].

In another study using semaglutide in a db/db mouse model (homozygous leptin receptor-deficient mice) of T2DM over 24 weeks, a reduction in liver injury, improvement in glucose metabolism, and decreased lipid levels were observed in the semaglutide-treated groups. Semaglutide was found to enhance gut barrier integrity and modulate the gut microbiota, increasing the abundance of *Alloprevotella* and *Alistipes* spp., while strains of *Ligilactobacillus* and *Lactobacillus* decreased, concurrently improving MASLD. Available data suggest that *Alloprevotella* and *Alistipes* produce SCFAs, which may partly contribute to their anti-inflammatory and cholesterol-lowering effects [91]. Conversely, in untreated mice, significant liver damage was observed, with pronounced dyslipidemia and elevated biochemical markers such as AST, ALT, and GGT [91].

The available preclinical and clinical data support the gut microbiome as a possible mediator of the hepatic effects of GLP-1-based therapies, although causality remains to be established in prospective human studies.

### 5.4. Gut Microbiome and Personalized Medicine

Increasing evidence suggests that the gut microbiome may play a role not only as a mediator of the therapeutic effects of GLP-1 receptor agonists but also as a potential predictor of treatment response. Tsai et al. (2022) [97] found that patients responding to GLP-1 agonists differed from non-responders in the beta diversity of their gut microbiome, suggesting the possibility of microbiome-based stratification prior to therapy initiation in patients with MASLD and concomitant T2DM.

Beyond its prognostic role, the microbiome may also serve as a therapeutic target. Niu et al. [98] demonstrated that liraglutide partially restores microbial diversity in patients with T2DM, approaching levels observed in healthy individuals—a promising therapeutic effect that could contribute to long-term metabolic improvements.

The mechanistic link between the microbiome and GLP-1RA therapy is supported by recent preclinical data. Gao et al. [96] showed that in db/db mice, semaglutide alone induces only moderate improvements in hepatic steatosis and glycemic control, with effects diminishing over time. The addition of *Akkermansia muciniphila* (strain Akk11) to semaglutide resulted in a synergistic effect, significantly enhancing the reduction in visceral fat, hepatic steatosis, serum triglycerides, and inflammatory markers. Akk11 is a specific strain of *Akkermansia muciniphila*—a pasteurized (non-viable) derivative used as a probiotic. Importantly, although it is pasteurized and cannot colonize the gut, it retains its bioactive components (including the outer membrane protein Amuc_1100), which mediate its metabolic and anti-inflammatory effects [99,100]. The combination remodels the gut microbiome, suppresses fatty acid synthesis, promotes mitochondrial function, and attenuates pro-inflammatory pathways in both the gut and liver. These findings provide proof-of-concept for microbiome-based adjuvant strategies in MASLD and suggest a stratified therapeutic approach. It could be speculated that patients with a favorable microbiome may respond well to GLP-1RA monotherapy, whereas patients with dysbiosis (e.g., low levels of *A. muciniphila*) may require a combined intervention including specific probiotics, prebiotics, or dietary modifications that promote the growth of beneficial microbial species. If baseline dysbiosis contributes to inadequate response to GLP-1RA, this may explain why some patients are “non-responders” in clinical trials and opens possibilities for personalized, microbiome-based therapy in MASLD.

A recently published clinical study by Klemets et al. (2026) [101], including 20 patients with T2DM, provided the first longitudinal data on the effect of semaglutide on the human gut microbiome. Results showed that semaglutide did not induce statistically significant changes in microbial diversity, suggesting that microbiome effects are more likely indirect, mediated through weight reduction and improved glycemic control rather than a direct pharmacological action on the microbial community. Nevertheless, baseline microbial profiles correlated with changes in HbA1c, supporting the concept of microbiome-based stratification prior to therapy [101]. These findings highlight the need for validation in larger cohorts with metagenomic sequencing, including patients with MASLD and MASH, to clarify causal relationships and identify reliable microbial biomarkers for personalized therapy selection. Key clinical studies are summarized in Table 2.

## 6. Discussion

This review summarizes the growing evidence on the role of GLP-1-based therapies in modulating the gut microbiome and their potential application in treating MASLD in patients with T2DM. Despite promising results from preclinical studies, several key aspects require further in-depth discussion.

### 6.1. Mechanistic Link Between Incretins and the Gut Microbiome

Data from animal models consistently show that GLP-1RAs and dual GLP-1/GIP agonists modulate the composition of the gut microbiome by normalizing the *Firmicutes*/*Bacteroidetes* ratio and increasing beneficial strains such as *Akkermansia muciniphila*, *Bifidobacterium*, and *Lactobacillus* spp. [55,81]. These changes correlate with improvements in metabolic parameters, reduction in hepatic steatosis, and decreased inflammation. However, it remains unclear whether the observed effects are a direct consequence of the pharmacological action of GLP-1-based therapies on the microbiome or an indirect result of reduced body weight, improved glycemic control, and dietary changes in treated animals.

Particularly interesting is the mechanism of action of tirzepatide, which not only induces changes in microbial composition but also modulates bile acid metabolism [81]. This suggests a complex bidirectional communication between GLP-1/GIP signaling, the gut microbiome, and liver function. The observed increase in short-chain fatty acid (SCFA) production during semaglutide treatment [93] supports the hypothesis of microbiome-mediated anti-inflammatory effects, which may contribute to MASLD improvement. It should also be noted that GLP-1RA-induced delayed gastric emptying and reduced intestinal motility may theoretically exert negative effects on gut microbiome composition, potentially promoting SIBO or altering microbial transit time; however, the clinical significance of these effects remains to be established in dedicated studies.

### 6.2. Gut–Liver Axis as a Therapeutic Target

The documented effects of GLP-1-based therapies on gut barrier integrity, bile acid metabolism, and SCFA production suggest that the gut–liver axis represents an important therapeutic target. Reduced intestinal permeability leads to lower LPS translocation and decreased hepatic inflammation. Increased SCFA production, particularly propionate, suppresses gluconeogenesis via activation of AMPK and GPR43 signaling pathways in vitro [48,111]. In combination with other SCFAs (acetate, butyrate), propionate may modulate lipid metabolism through receptor-mediated mechanisms and improve insulin sensitivity. However, in vivo human data suggest that propionate effects may be context-dependent and influenced by host metabolic status.

These mechanisms likely act synergistically with the direct effects of GLP-1-based therapies (GLP-1RA, GIP/GLP-1 co-agonists) on hepatic metabolism and weight reduction in MASLD/MASH and type 2 diabetes, resulting in decreased hepatic steatosis, improved insulin resistance, and reduced inflammation [6,112]. The effects of GLP-1 receptor agonists on individual lipid parameters and their relationship with gut microbiota modulation are summarized in Table 3.

GLP-1-based therapies primarily reduce de novo lipogenesis indirectly through weight loss, reduced adiposity, and improved glycemic control. At the molecular level, GLP-1 receptor agonists directly suppress hepatic lipogenesis through downregulation of SREBP-1c, FASN, and DGAT1, and reduce hepatic ApoB expression [117]. Liraglutide additionally decreases intestinal expression of ApoB48, DGAT1, and MTP, thereby limiting intestinal lipoprotein assembly [117]. These effects are complemented by microbiota-mediated mechanisms: GLP-1 receptor agonist-induced enrichment of *Faecalibacterium prausnitzii* correlates negatively with glycemia and improves lipid profile, while shifts toward SCFA-producing taxa further modulate lipoprotein metabolism [83,107]. GLP-1 agonists increase LPL expression and decrease PCSK9 expression in adipose tissue, enhancing lipoprotein catabolism [120]. The clinical relevance of these findings is underscored by the observation that attenuation of postprandial lipemia may represent one of the key mechanisms underlying the cardiovascular benefits of this drug class, and that integration of dietary interventions with GLP-1 receptor agonist therapy may further optimize hepatic outcomes through modulation of the gut microbiota and GLP-1 signaling axis.

### 6.3. Clinical Significance and Future Perspectives

FDA approval of semaglutide for the treatment of MASH with fibrosis stages F2 and F3, based on the ESSENCE trial [11], represents a major step in the therapeutic application of GLP-1-based therapies in liver disease. The impressive results, showing a 62.9% reduction in inflammation without worsening or even with regression of fibrosis, highlight the clinical potential of this drug class. However, the role of gut microbiome modulation in this beneficial effect remains unclear. Future studies should focus on several key areas. Firstly, clinical trials in humans are needed that include comprehensive gut microbiome analyses via metagenomic sequencing, as well as metabolomic and metaproteomic approaches. This would allow identification of specific microbial signatures that correlate with therapeutic response and could serve as predictive biomarkers. Secondly, investigating combination therapeutic strategies, including GLP-1-based drugs and targeted dietary interventions (prebiotics, probiotics, synbiotics, postbiotics), could optimize gut microbiome modulation and enhance beneficial metabolic effects. Particularly promising are novel triple agonists of GLP-1/GIP/glucagon receptors such as retatrutide, as well as combinations of GLP-1 RA with amylin analogs, which show even more pronounced weight loss and metabolic improvements [41]. The molecular mechanisms through which GLP-1-based therapies influence the microbiome—whether via direct action on the gut epithelium and immune system, changes in intestinal motility and secretion, or central mechanisms affecting feeding behavior—need clarification. Animal models and fecal transplantation experiments could provide valuable insights into causal relationships.

Despite existing limitations, the accumulated evidence provides a solid foundation for further investigation of the link between GLP-1-based therapies, the gut microbiome, MASLD and MASH. Integration of microbiome analyses into clinical practice could improve patient stratification and allow identification of individuals who would benefit the most from this therapeutic strategy.

## 7. Limitations and Challenges

This review has several limitations that should be considered when interpreting the results. First, as a narrative review, it does not include a quantitative synthesis of the data and is potentially subject to publication bias, since positive results are more likely to be published than negative or neutral findings. The heterogeneity of the included studies—different animal models, doses, treatment durations, and microbiome analysis methods—makes direct comparison across studies challenging. A major limitation of the available data is the predominance of experimental studies in animal models, primarily rats and mice. Although similarities between the gut microbiome of rats and humans have been observed at the phylum level [69], there are significant differences in microbial diversity and interindividual variability, which limit the direct extrapolation of results to the human population. In addition, experimental models of diabetes induced by high-fat diet and streptozotocin do not fully recapitulate the complex pathophysiology of human T2DM and MASLD. Another key limitation is the short-term nature of most studies, which does not allow assessment of long-term effects on the microbiome and liver fibrosis. A clinical study with liraglutide in humans [79] demonstrated a relatively modest effect on gut microbiome composition, despite improvements in metabolic parameters and liver function. This raises the question of whether microbiome changes are a primary mechanism of action in humans or rather a secondary effect. The most recent clinical study by Klemets et al. (2026) [101] in patients with T2DM provided important data on the effect of semaglutide on the human gut microbiome. Although the results showed that semaglutide did not induce statistically significant changes in microbial diversity, this suggests that microbiome effects are more likely indirect, mediated by weight reduction and improved glycemic control, rather than a direct pharmacological effect on the microbial community [101].

It is important to note that modulation of the gut microbiome should not be considered an isolated therapeutic mechanism but as part of the complex multifactorial effects of GLP-1-based therapies. Weight reduction, improved glycemic control, decreased insulin resistance, and direct effects on hepatic metabolism likely contribute synergistically to the overall therapeutic outcome. Distinguishing the individual contribution of each of these mechanisms remains a challenge for future research. The high interindividual variability of the human gut microbiome suggests that therapeutic responses may differ substantially depending on a patient’s baseline microbial profile. This underscores the need for a personalized approach in MASLD treatment that accounts not only for traditional clinical parameters but also for individual gut microbiome characteristics.

## 8. Conclusions

Management of T2DM, MASLD, and MASH requires a comprehensive, integrated approach that combines lifestyle modifications with pharmacological therapy aimed at weight regulation and reduction in metabolic risk factors. Available preclinical data demonstrate that GLP-1 receptor agonists liraglutide and semaglutide, as well as the dual GLP-1/GIP agonist tirzepatide modulate the gut microbiome through multiple interconnected mechanisms. These changes correlate with reductions in hepatic steatosis and associated inflammation, confirming the therapeutic potential of this class of drugs in metabolic dysfunction-associated liver disease. Nevertheless, the extent to which these microbiome changes directly mediate hepatic outcomes in humans remains to be prospectively established. This review provides a timely synthesis of this rapidly evolving evidence, incorporating the most recent clinical milestones including the FDA approval of semaglutide for MASH with fibrosis. It introduces the concept of microbiome-based stratification as a foundation for personalized therapy in MASLD, supported by emerging data demonstrating that baseline microbiome composition may predict glycemic response to GLP-1-based treatment. Future prospective clinical trials integrating comprehensive gut microbiome analyses are warranted to elucidate causal mechanisms and identify microbial biomarkers that could guide treatment selection in patients with MASLD and MASH.

## Figures and Tables

**Table 1:** Preclinical studies on the effects of GLP-1-based therapies on gut microbiome and liver function.

Author	Model	Drug	Effect on Microbiome	Effect on Liver
Wang et al., 2016 [83]	C57BL/6 Mice (n = 60)	Liraglutide 0.4 mg/kg s.c. daily vs. saxagliptin 10 mg daily 8 weeks	In liraglutide groups, 13 taxa increased (incl. *Allobaculum*, *Turicibacter*, *Anaerostipes*, *Blautia*, *Lactobacillus*, *Desulfovibrio*) and 20 decreased (*Clostridiales* and *Bacteroidales*, *Roseburia*, *Marvinbryantia*) No significant changes in saxagliptin groups	Not assessed
Wang et al., 2017 [84]	Mice (DIO)	Exenatide 10 μg/kg two times daily for 28 days	Not assessed	Reduced hepatic steatosis and oxidative stress; improved insulin resistance, mitochondrial function and respiratory chain
Madsen et al., 2019 [85]	Mice (DIO)	Liraglutide (0.2 mg/kg BID, 4 weeks)	Phylogenetically similar changes in gut bacterial composition; discrete changes in low-abundance species and associated bacterial metabolic pathways	Not assessed
Kalavalapalli et al., 2019 [86]	Mice C57BL/6J (TFD diet, 24 weeks)	Exenatide (30 μg/kg/day) 8 weeks treatment	Not assessed	Reduced hepatic glucose production, pyruvate cycling (17%); decreased intrahepatic TG content (31%); reduced diacylglycerols and ceramides; decreased Srebp1C, Cd36, Tnfa, Timp1 expression
Liu et al., 2020 [87]	Mice (db/db vs. wt/wt) n = 40	Liraglutide 0.2 mg/g intraperitoneally (i.p.) 28 days	Increased *Akkermansia*, *Romboutsia*, Bacteroidales group; decreased *Klebsiella*, *Anaerotruncus*, *Bacteroides*, *Lachnospiraceae*	Significantly reduced hepatic TG content and ALT activity; improved hepatic steatosis
Zhang et al., 2020 [55]	Rats Groups (n = 24): NC (n = 8) saline s.c. HFD (n = 8) saline s.c. HFD + liraglutide (n = 8)	Liraglutide 0.2 mg/kg/day s.c. for 4 weeks	Normalization of *Firmicutes*/*Bacteroidetes* ratio; increased *Bifidobacterium* and *Lactobacillus* spp.	Not assessed
Saad et al., 2020 [88]	Rats Wistar (n = 30, 6 groups) HFD	Exenatide (10–40 μg/kg/day s.c. 7 weeks of treatment	Not assessed	Improved hyperglycemia, hyperinsulinemia, liver enzymes, hypertriglyceridemia; reduced hepatic lipid peroxides and inflammatory mediators (IL-6, NF-kB, TNF-a, TLR4); attenuated hepatic fatty degeneration
Niu et al., 2022 [89]	Mice C57BL/6J (HFD-induced NAFLD) n = 24	Semaglutide 30 μmol/kg i.p. once daily for 12 weeks	Not assessed	Reduced body weight, hepatic weight, blood glucose, TG, TC, LDL; decreased pro-inflammatory factors; improved hepatocyte steatosis and ballooning degeneration
Zhao et al., 2022 [78]	C57BL/6 Mice n = 24	Normal control (n = 8) HFD + saline s.c. (n = 8) HFD + Liraglutide 0.2 mg/kg s.c. daily for 12 weeks (n = 8)	Reduced *Firmicutes*/*Bacteroidetes* ratio in liraglutide-treated group; increased *Akkermansia*, *Lactobacillus*, *Parabacteroides*, *Oscillospira*; decreased *Shigella*, *Proteobacteria*. Greatest increase in *Akkermansia*	Not assessed
Pontes-da-Silva et al., 2022 [90]	Mice C57BL/6 (HFD 16 weeks)	Semaglutide (40 μg/kg) 4 weeks of treatment	Not assessed	Reduced hepatic steatosis; improved hormones and adipokines; decreased lipogenesis and inflammation; increased beta-oxidation; reduced hepatic glucose uptake and ER stress
Tuohua Mao et al., 2024 [91]	Mice (db/db)	Groups: db/m − saline i.p.; db/db saline i.p.; db/db + Semaglutide 0.22 mg/kg i.p. every 3 days Duration: 16 weeks treatment (24 weeks total)	Increased *Alloprevotella* and *Alistipes*; decreased *Lactobacillus*	Reduction in liver injury; improved MASLD; decreased ALT, AST, GGT
Duan et al., 2024 [92]	Mice C57BL/6J (HFD, 10 weeks)	Semaglutide 18 days treatment	Attenuated HFD-induced microbial dysbiosis; restored *Akkermansia*, *Faecalibaculum*, *Allobaculum*; decreased *Lachnospiraceae*, *Bacteroides*; increased tight junction proteins	Reduced hepatic lipid deposition and fat droplet formation; regulated expression of genes related to abnormal blood glucose
Hu et al., 2025 [81]	C57BL/6J mice; HFD + STZ-induced T2DM (n = 56)	NC (n = 8) standard diet + PBS i.p. MC (n = 8) HFD/STZ + PBS i.p. MC + Semaglutide (n = 8) 30 nmol/kg/day i.p. MC + Tirzepatide (n = 8) 10 nmol/kg/day i.p 12 days role of gut microbiota, (n = 24): In Parallel: MC (n = 8) + PBS MC + Tirzepatide 10 nmol/kg/day i.p. (n = 8) MC + Abx + Tirzepatide (n = 8) for 14 days	Both tirzepatide and semaglutide remodeled gut microbiota in HFD/STZ diabetic mice. Tirzepatide notably increased *Akkermansia (Verrucomicrobiota)* and showed an upward trend in *Ligilactobacillus* and *Dubosiella*; semaglutide increased *Lactobacillus*, *Limosilactobacillus (Firmicutes)* and *Akkermansia*. Both treatments decreased *Erysipelatoclostridium*. Tirzepatide showed superior enhancement of *Akkermansia* compared to semaglutide. *Akkermansia* and *Lactobacillus* negatively correlated with FBG, insulin resistance, and hepatic lipid parameters.	Tirzepatide reduced liver weight, liver index, hepatic TC and TG, lipid droplet area, and serum TC, TG, ALT and AST. Semaglutide reduced serum ALT and AST but did not significantly reduce serum TC and TG. Tirzepatide showed superior efficacy in reducing lipid accumulation compared to semaglutide.
Liang J. et al., 2025 [82]	Mice (n = 29)	NCD (n = 10) standard diet + placebo (Tris-HCl buffer) s.c.; HFD (n = 9) HFD + placebo s.c.; HFD + Tizepatide (n = 10) dose escalation s.c. twice weekly: 0.03 mg/kg (weeks 1–4); 0.1 mg/kg (weeks 5–8); 0.3 mg/kg (weeks 9–12) 12 weeks of treatment	Not assessed	Reduction in ALT, AST, TG, TC; improved mitochondrial–lysosomal function; increased cholesterol efflux
Luo Y. et al., 2025 [93]	C57BL/6 mice (n = 21), 5 weeks old; HFD (4 weeks) + STZ 40 mg/kg i.p. × 4 days; T2DM confirmed by FBG > 11.1 mmol/L	Control (n = 5) standard diet; T2DM (n = 8) HFD + STZ; T2DM + Semaglutide (n = 8) HFD + STZ + semaglutide 40 μg/kg s.c. every 3 days for 4 weeks	Decreased *Firmicutes*, *Actinobacteriota*, *Lactobacillus*; increased *Bacteroides*, *Muribaculaceae*, and SCFA	Not assessed
Wang et al., 2025 [94]	Mice C57BL/6J (n = 40) HFD, 10 weeks	Tirzepatide 14 days treatment	Alleviated HFD-induced dysbiosis; restored *Akkermansia*, *Bacteroides*, *Mucispirillum*, *Enterococcus*, *Alistipes; decreased Faecalibaculum*, *Allobaculum*, *Ileibacterium*	Attenuated lipid deposition and fat droplet formation; suppressed weight gain; improved insulin resistance
Sun et al., 2025 [95]	Mice C57BL/6J (n = 50) HFD	ND (n = 10) standard diet + saline i.p. every other day; HFD (n = 10) HFD + saline i.p. every other day; LSHF (n = 10) HFD + semaglutide 10 μg/kg i.p. every other day; MSHF (n = 10) HFD + semaglutide 40 μg/kg i.p. every other day; HSHF (n = 10) HFD + semaglutide 100 μg/kg i.p. every other day	Remodeled fecal microbiota composition and proportion; effects on metabolic pathways (amino acid metabolism, pyrimidine metabolism)	Reduced body weight, body fat, FBG and insulin levels; improved insulin resistance and sensitivity; regulated lipid metabolism gene expression
Gao et al., 2026 [96]	Mice (db/db)	Control normal diet + saline; db/db negative control normal diet + saline; db/db + Semaglutide 40 μg/kg s.c. every 3 days; db/db + Semaglutide + Akk11 semaglutide 40 μg/kg i.p. every 3 days + Akk11 2 × 10^8^ CFU oral gavage every 2 days for 14 days	Semaglutide monotherapy: Restored *Firmicutes*/*Bacteroidetes* ratio (reversed diabetes-induced dysbiosis); restored *Akkermansia muciniphila* abundance toward WT levels; decreased *Muribaculaceae*; Semaglutide + *Akkerrmasia:* synergistic remodeling of gut microbiota; upregulated intestinal amino acid transporters; increased ZO-1 expression (improved gut barrier integrity)	Significant reduction in visceral fat, hepatic steatosis, and inflammatory markers

Abbreviations: ALT—alanine aminotransferase; AST—aspartate aminotransferase; TG—triglycerides; TC—total cholesterol; GGT—gamma-glutamyl transferase; SCFA—short-chain fatty acids; HFD—high-fat diet; STZ—streptozotocin; MASLD—metabolic dysfunction-associated steatotic liver disease; db/db mice—homozygous leptin receptor-deficient mice; db/m—heterozygous littermate controls; C57BL/6J—inbred wild-type mice; CFU—colony-forming unit; ND—normal diet; LSHF—low-dose semaglutide + HFD; MSHF—medium-dose semaglutide + HFD; HSHF—high-dose semaglutide + HFD, ZO-1—zonula occludens.

**Table 2:** Summary of recent clinical studies reporting the effects of GLP-1 receptor agonists on the microbiome and the liver.

Author/Study	Population	Drug	Effect on Microbiome	Effect on Liver
Armstrong et al., 2016 [102] (LEAN trial)	Patients with NASH (n = 52)	Liraglutide 1.8 mg s.c. once daily 48 weeks	Not evaluated	Resolution of NASH in 39% vs. 9% in placebo
Cusi et al., 2018 (AWARD programme) [103]	T2DM patients (n = 1499)	Dulaglutide 1.5 mg (n = 971) vs. placebo (n = 528)	Not evaluated	At 6 months: significantly reduced ALT (−1.7 IU/L, *p* = 0.003), AST (−1.1 IU/L, *p* = 0.037), GGT (−6.6 IU/L, *p* = 0.025); in NAFLD/NASH subgroup: more pronounced ALT reductions
Shang et al., 2021 [104]	T2DM patients (n = 40)	Liraglutide 1.2 mg s.c. once for daily 4 months	Reduced alpha diversity; altered community structure; 21 species enriched before treatment, 15 species after treatment;	Not evaluated
Smits et al., 2021 [105]	Adults with T2DM (n = 51)	Liraglutide 1.8 mg s.c. daily vs. sitagliptin 100 mg vs. placebo (12 weeks)	Neither liraglutide nor sitagliptin affected alpha or beta diversity of the gut microbiome; changes in microbial composition were not associated with clinical parameters	Liraglutide reduced HbA1c by 1.3%, trend toward body weight reduction (1.7 kg); increased fecal secondary bile acid deoxycholic acid
Newsome et al., 2021 [106]	NASH patients (n = 320, F1–F3)	Semaglutide 0.1, 0.2, 0.4 mg daily s.c. or placebo (72 weeks)	Not evaluated	NASH resolution: 59% in treatment group vs. 17% placebo (*p* < 0.001); dose-dependent reduction in fibrosis progression, but no statistically significant improvement in fibrosis stage
Tsai et al., 2022 [97]	Patients with MASLD + T2DM	GLP-1RAs	Differences in beta diversity between responders and non-responders	Not evaluated
Niu et al., 2023 [98]	Patients with T2DM	Liraglutide	Partial restoration of microbial diversity	Not evaluated
Ying et al., 2023 [79]	Patients with T2DM (n = 15)	Liraglutide 1.8 mg daily vs. Metformin 1500 mg daily for 12 weeks	Liraglutide significantly increased the diversity and richness of the gut bacterial community; increased relative abundance of *Bacteroidetes*, *Proteobacteria*, *Bacilli*	Improved liver function; reduction in body weight and plasma glucose
Liang et al., 2023 [107]	T2DM patients (n = 12)	GLP-1 RAs (1 week)	Significantly increased abundance of *F. prausnitzii* (*p* = 0.002); significant negative correlation with fasting glucose no change in *L. delbrueckii*	Not evaluated
Zheng et al., 2023 [108]	T2DM patients (n = 71)	Dulaglutide 0.75 mg s.c. for 4 weeks + 1.5 mg/week (20 weeks) + standard treatment (metformin, sulphanylurea and/or insulin) (n = 25) vs. standart treatment only (n = 46)	Not evaluated	Greater reduction in hepatic fat, pancreatic fat, and liver stiffness (*p* <0.001); significant improvements in liver function tests, renal function tests, lipid profiles, and blood parameters
Loomba et al., 2024 [109] (SYNERGY-NASH)	Patients with MASH	Tirzepatide 5,10,15 mg s.c. once weekly for 52 weeks	Not evaluated	Resolution of MASH in 44–62% vs. 10% in placebo
Michel & Schatenberg, 2025 (ESSENCE) [10]	Patients with MASH + fibrosis F2–F3 (n = 800)	Semaglutide 2.4 mg s.c. for 72 weeks	Not evaluated	62.9% resolution of MASH without fibrosis worsening
Arai et al., 2025 [110]	MASLD + T2DM patients (n = 13)	Tirzepatide 2.5 mg once weekly for 4 weeks; dose adjustments based on efficacy and adverse events (48 weeks)	Not evaluated	Significant improvements in body weight, liver enzymes, and HbA1c at week 12, sustained to week 48;
Chen et al., 2025 [80]	Patients with T2DM (n = 15)	Semaglutide 0.25 mg titrated up to 1 mg weekly for 12 weeks	Decrease in *Firmicutes*; increase in *Bacteroidota*, *Actinobacteriota* and *Bifidobacterium*	Not evaluated
Klemets et al., 2026 [101]	Patients with T2DM (n = 20)	Semaglutide 0.25 mg t to 1 mg weekly or Empagliflozin * 10 mg orally once daily 12 months	The effects on the microbiome are more likely indirect (due to improvements in metabolic health, baseline microbial profile correlated with changes in HbA1c)	Not evaluated

Abbreviations: T2DM—type 2 diabetes mellitus; NASH—non-alcoholic steatohepatitis; MASH—metabolic-associated steatohepatitis; MASLD—metabolic dysfunction-associated steatotic liver disease; GLP-1RA—glucagon-like peptide-1 receptor agonist; HbA1c—glycated hemoglobin; * Klemets et al. (2026) [101] included an empagliflozin arm as an active comparator; empagliflozin is an SGLT-2 inhibitor.

**Table 3:** Effects of GLP-1 receptor agonists on lipid parameters and their relationship with gut microbiota.

Lipid Parameter	Effect of GLP-1 Agonists	Relationship with Gut Microbiota	References
LDL-C	Modest reduction, independent of weight loss; semaglutide shows best effect	Gut microbiota modulates bile acid metabolism through FXR signaling, increasing hepatic LDL receptor expression and enhancing LDL clearance; TGR5 contributes indirectly through anti-inflammatory effects and improved metabolism; GLP-1 agonists have been shown to alter microbial composition leading to improved lipid profile	[75,113,114,115]
TC	Reduction of TC; semaglutide shows significant reduction	Microbiome changes induced by GLP-1 agonists (increased *Akkermansia muciniphila*, *Faecalibacterium prausnitzii*) correlate with improved total cholesterol metabolism through reduced endotoxemia and increased SCFA production	[75,85,113,114]
TG	Modest reduction; semaglutide showed significant reduction in postprandial triglycerides)	Gut microbiota-derived SCFAs stimulate GLP-1 secretion via FFAR2/FFAR3 receptors on enteroendocrine L-cells; circulating SCFAs are negatively associated with triglycerides and non-esterified fatty acids in humans; GLP-1 agonists reciprocally enrich SCFA-producing taxa, creating a bidirectional metabolic loop that reduces triglyceride synthesis and improves lipid metabolism	[75,114,116,117,118,119]
VLDL-C	Reduction in atherogenic lipoproteins; GLP-1 agonists inhibit VLDL production and increase VLDL-apoB100 catabolism	Gut dysbiosis increases intestinal permeability and metabolic endotoxemia, promoting hepatic VLDL overproduction; GLP-1 agonists restore gut barrier integrity and reduce VLDL secretion	[85,113,115,120]
HDL-C	Inconsistent effects; most studies show no significant increase	Microbial metabolites (SCFAs, secondary bile acids) may indirectly influence HDL metabolism through improved insulin sensitivity and reduced systemic inflammation	[113,114,115]
NEFA	Significant reduction; inhibition of adipose lipolysis through increased insulin and decreased glucagon;	Gut microbiota-derived SCFAs and bile acids modulate adipose lipolysis and hepatic fatty acid oxidation through AMPK activation; GLP-1 agonists enhance β-oxidation and inhibit adipose lipolysis through increased insulin and decreased glucagon, reducing circulating NEFA.	[116,121,122,123]
CM	Reduced postprandial chylomicron production from enterocytes; significant blunting of postprandial triglyceride and ApoB48 rise; liraglutide reduces ApoB48 production by 35–60% through downregulation of intestinal ApoB48, DGAT1, and MTP expression	Gut microbiota regulates intestinal lipid absorption and chylomicron production through modulation of epithelial lipid digestion and transport; *Lactobacillus paracasei* inhibits chylomicron secretion via L-lactate/malonyl-CoA, while *E. coli* promotes lipid oxidation through acetate-mediated AMPK/PGC-1α/PPARα activation	[116,120,124,125]
Hepatic lipids	Significant reduction in hepatic triglyceride and cholesterol content in preclinical models; MASH resolution in 59–62.9% of patients in clinical trials (ESSENCE, SYNERGY-NASH)	Gut microbiota-derived SCFAs reduce hepatic triglyceride and cholesterol accumulation through AMPK activation and induction of fatty acid oxidation genes; SCFAs modulate AMPK/SIRT1 signaling, improving insulin sensitivity and reducing hepatic inflammation; GLP-1 agonists restore gut–liver axis functionality, with effects enhanced through dietary modulation of the microbiota SCFA-GLP-1 pathway	[10,57,75,109,123]

Abbreviations: LDL-C—low-density lipoprotein cholesterol; HDL-C—high-density lipoprotein cholesterol; VLDL-C—very low-density lipoprotein cholesterol; NEFA—non-esterified fatty acids; ApoB48—apolipoprotein B48; SCFA—short-chain fatty acids; FXR—farnesoid X receptor; TGR5—G protein-coupled bile acid receptor; AMPK—AMP-activated protein kinase; SIRT1—sirtuin 1; DGAT1—diacylglycerol acyltransferase 1; CM—chylomicrons; FFAR—free fatty acid receptor; MTP—microsomal triglyceride transfer protein.

## Data Availability

No new data were created or analyzed in this study.

## Abbreviations

ALD	alcoholic liver disease
ALT	alanine aminotransferase
AMPK	AMP-activated protein kinase
AST	aspartate aminotransferase
DAMPs	damage-associated molecular patterns
FABP4	Fatty acid-binding protein 4
FGF	fibroblast growth factor
FXR	farnesoid X receptor
GCGR	glucagon receptor
GGT	gamma-glutamyl transferase
GIP	glucose-dependent insulinotropic polypeptide
GIT	gastrointestinal tract
GLP-1	glucagon-like peptide-1
GLP-1RA	GLP-1 receptor agonist
GPR43	G-protein-coupled receptor 43
HbA1c	glycated hemoglobin
HDL	high-density lipoprotein
HFD	high-fat diet
IL	interleukin
IRF3	interferon regulatory factor 3
LPS	lipopolysaccharide
MASH	metabolic dysfunction-associated steatohepatitis
MASLD	metabolic dysfunction-associated steatotic liver disease
MyD88	myeloid differentiation primary response 88
NAFLD	non-alcoholic fatty liver disease
NASH	non-alcoholic steatohepatitis
NLRP3	NOD-, LRR-, and pyrin domain-containing protein 3
PPAR	peroxisome proliferator-activated receptor
SCFA	short-chain fatty acids
SIBO	small intestinal bacterial overgrowth
SREBP	sterol regulatory element-binding protein
STZ	streptozotocin
T2DM	type 2 diabetes mellitus
TC	total cholesterol
TG	triglycerides
TGR5	G-protein-coupled bile acid receptor
TLR4	Toll-like receptor 4
TNF-α	tumor necrosis factor alpha
UCP2	uncoupling protein 2
